# Gastrointestinal Seed Bezoars: A Systematic Review of Case Reports and Case Series

**DOI:** 10.7759/cureus.4686

**Published:** 2019-05-17

**Authors:** Dimitrios K Manatakis, Vasileios Acheimastos, Maria Ioanna Antonopoulou, Dimitrios Balalis, Dimitris P Korkolis

**Affiliations:** 1 Surgery, Athens Naval and Veterans Hospital, Athens, GRC; 2 Surgery, Saint Savvas Cancer Hospital, Athens, GRC

**Keywords:** bezoar, phytobezoar

## Abstract

Seed bezoars are a distinct subcategory of phytobezoars, caused by indigestible vegetable or fruit seeds. The aim of our study was to present a comprehensive review on seed bezoars, focusing on epidemiology, symptomatology, diagnosis and treatment options.

A systematic review of the English literature (1980-2018) was conducted, using PubMed, Embase and Google Scholar databases. Fifty-two studies fulfilled the inclusion criteria, with a total of 153 patients, the majority of whom (72%) came from countries around the Eastern Mediterranean and the Middle East.

Patients complained primarily about constipation (63%), abdominal/rectal pain (19%) or intestinal obstruction (17%). Most seed bezoars were found in the rectum (78%) and the terminal ileum (16%). Risk factors were recognised in 12% of cases. Manual disimpaction under general anaesthesia was the procedure of choice in 69%, while surgery was required in 22% of cases.

Seed bezoars appear to represent a different pathophysiological process compared to fibre bezoars. Seeds usually pass through the pylorus and ileocaecal valve, due to their small size, and accumulate gradually in the colon. Seed bezoars are usually found in the rectum of patients without predisposing factors, causing constipation and pain. History and digital rectal examination are the mainstay of diagnosis, with manual extraction under general anaesthesia being the procedure of choice.

## Introduction and background

Bezoars are retained aggregates of indigestible material that accumulate and conglomerate in the gastrointestinal tract. They can occur anywhere from the oesophagus to the rectum, however, they are most commonly found in the stomach. Recognised risk factors are prior gastric surgery, neuropsychiatric disease, endocrinopathies impairing gastrointestinal motility and poor mastication [1, 2].

Based on their component, they are classified into four main types: phytobezoar (fruit and vegetable fibres), trichobezoar (hair), lactobezoar (undigested milk concretions) and pharmacobezoar (medications) [1, 2]. Seed bezoars are a distinct subtype of phytobezoars, caused by undigested vegetable seeds or fruit pits. In contrast to other bezoar categories, the majority is found in the rectum of patients with no predisposing factors, a fact suggesting a different pathophysiological process [3, 4].

We hereby present a systematic, up-to-date review of case reports and case series of gastrointestinal seed bezoars, with emphasis on epidemiology, diagnosis and treatment options.

## Review

Methods

We performed a systematic review of the literature, following the Meta-Analysis of Observational Studies in Epidemiology (MOOSE) guidelines, in order to identify all studies of patients with gastrointestinal seed bezoars [5]. Literature searches were conducted in PubMed/MEDLINE, EMBASE and Google Scholar bibliographic databases, spanning years 1980 to 2018. The keywords “bezoar”, “phytobezoar”, “seed”, “grain” and “pit” were used in all possible combinations. Moreover, the reference lists of all eligible studies were assessed for additional articles.

All study designs (case series and case reports) were eligible for inclusion in the final analysis. Patient age was not an exclusion criterion, and both adult and paediatric cases were included in the review. Articles without full text availability were excluded.

**Figure 1:**
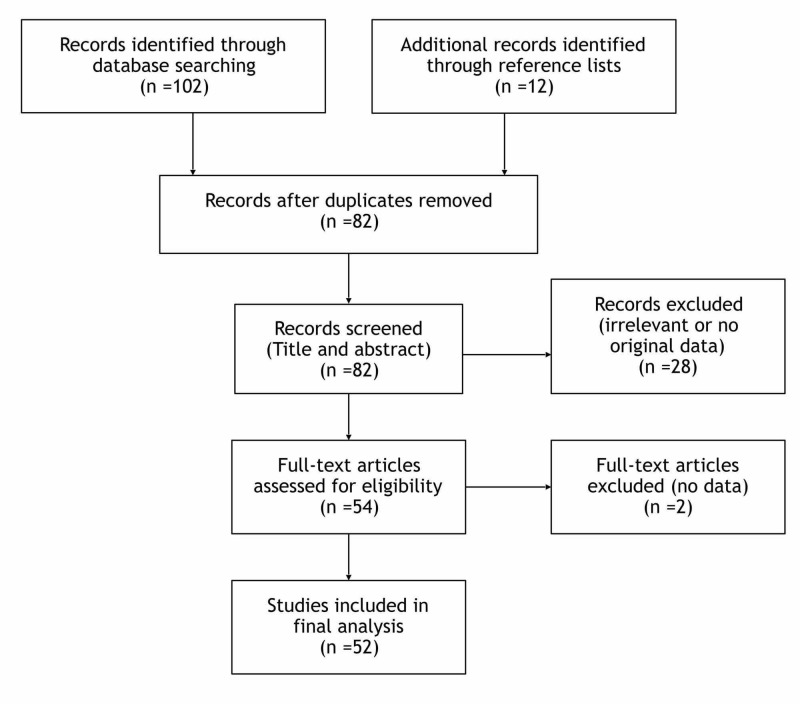
Flowchart of literature search

Titles and abstracts of all articles from the initial search were independently screened by two authors, to determine those articles for full text review. Any discrepancies concerning the evaluation of the studies were arbitrated by all authors. A flow chart of study selection is shown in Figure 1.

For each eligible study, data were extracted about demographic (number of patients, age, sex, and country of origin) and clinical characteristics (seed type, predisposing factors, bezoar location, clinical presentation, diagnostic and therapeutic management).

Statistical analysis was performed on SPSS, version 20.0 (IBM Corp, Armonk, NY). All data were tabulated and outcomes were cumulatively analysed. Continuous variables were expressed as mean ± standard deviation, while categorical variables were expressed as frequencies or percentages. Additionally, adult and paediatric subgroups were compared, using Student’s t-test for continuous variables and chi-square or Fisher’s exact test for categorical variables. Statistical significance was set to p < 0.05.

Results

Fifty-two studies were included in the final analysis (two retrospective case series and 50 case reports), with a total of 153 patients (Appendix). Demographic characteristics of patients are presented in Table 1.

**Table 1:** Demographic characteristics of seed bezoar cases

N	153
Gender	
Male	90
Female	63
Age range	2 – 80 years
Paediatric cases	2 – 16 years (median 10 years)
Adult cases	18 – 80 years (median 44 years)
Country of origin	
Eastern Mediterranean basin - Middle East	110 (71.9%)
Western Europe - America	29 (19.0%)
Asia-Oceania	14 (9.1%)

The vast majority of cases (110/153, 71.9%) came from countries of the eastern Mediterranean basin and the Middle East, whereas 29 patients (19.0%) came from Western countries and 14 (9.1%) from Asia.

As far as the type of seed is concerned, most patients (55/153, 36.0%) consumed watermelon seeds (45 children, 10 adults), followed by sunflower seeds (30/153, 19.6%, 18 children, 12 adults) and prickly pears (28/153, 18.3%, six children, 22 adults). Other cases involved wild banana seeds (11/153, 7.2%), pumpkin seeds (5/153, 3.3%), pomegranate seeds (4/153, 2.6%), date seeds (2/153, 1.3%), tangerine pits (2/153, 1.3%), olive pits (2/153, 1.3%), lupin seeds (2/153, 1.3%), pop corn kernels (2/153, 1.3%), freekeh grains, sesame seeds, Baccaurea macrocarpa seeds, jaboticaba seeds, peanuts, lentil seeds, cherry pits, box myrtle seeds, mango seeds and granadilla seeds (one case each, 0.65%).

The majority of patients presented at the emergency department complained of constipation (96/153, 62.7%, 57 children, 39 adults). Twenty-nine patients (19.0%, 17 children, 12 adults) reported atypical abdominal or rectal pain accompanied by tenesmus or blood-tinged stools. The diagnostic workup revealed intestinal obstruction in 26 cases (17.0%, nine children, 17 adults). One elderly patient was diagnosed with acute abdomen due to rectal perforation, while in one case the seed bezoar was an incidental intraoperative finding. Risk factors for bezoar formation were recognised in 18 cases (11.7%, one child, 17 adults) and included gastrointestinal strictures (10/153, 6.5%), diabetes (4/153, 2.6%) and neuropsychiatric disease (4/153, 2.6%).

A thorough history and digital rectal examination at the emergency room usually hinted at the diagnosis. Endoscopy was employed in 20 patients (13.1%) and radiology (X-rays or abdominal CT scans) in 20 patients (13.1%). Rectal bezoars were found in 119 cases (77.8%, 70 children, 49 adults), followed by bezoars in the terminal ileum in 25 cases (16.3%, 11 children, 14 adults). In three cases (1.9%) bezoars were located in the sigmoid colon, in two cases each (1.3%) in the stomach and jejunum, whereas in one case each (0.66%), in the duodenum and caecum. Treatment options included manual disimpaction (106/153, 69.3%, 65 children, 41 adults), surgery (33/153, 21.5%, 12 children, 21 adults), endoscopy (5/153, 3.3%, five adults) or conservative measures (9/153, 5.9%, six children, three adults) (Table 2).

**Table 2:** Clinical characteristics of seed bezoar cases

	N	%
Clinical presentation		
Constipation	96	62.7
Abdominal/rectal pain	29	19.0
Intestinal obstruction	26	17.0
Rectal perforation	1	0.65
Asymptomatic finding	1	0.65
Diagnostic workup		
Endoscopy	20	13.1
Radiology (X-rays, CT scans)	20	13.1
Treatment		
Manual disimpaction	106	69.3
Surgery	33	21.5
Endoscopy	5	3.3
Conservative measures	9	5.9

Comparison of paediatric and adult populations (Table 3) showed that gender did not differ significantly between the two groups (p = 0.09), whereas consumption of watermelon seeds was more popular among children and of prickly pears among adults (p < 0.0001).

**Table 3:** Comparison of paediatric versus adult cases

	Children	%	Adults	%	p-value
N	83		70		
Age (years)	2-16 (median 10)		18-80 (median 44)		
Gender					
Male	54	65.1	36	51.4	
Female	29	34.9	34	48.6	0.09
Seed type					
Watermelon	45	54.2	10	14.3	
Sunflower	18	21.7	12	17.1	
Prickly pear	6	7.2	22	31.4	<0.0001
Localisation					
Rectum	70	84.3	49	70	
Terminal ileum	11	13.3	14	20	0.17
Symptoms					
Constipation	57	68.7	39	55.7	
Abdominal/rectal pain	16	19.3	12	17.1	
Intestinal obstruction	9	10.8	17	24.3	0.07
Treatment					
Manual disimpaction	65	78.3	41	58.6	
Surgery	12	14.5	21	30	
Conservative/endoscopy	6	7.2	8	11.4	0.03
Risk factors	1	1.2	17	24.3	<0.0001

In both groups, the rectum was the most common location of the seed bezoar and constipation was the prevailing symptom. Intestinal obstruction was more frequent in adults than in children (24.3% vs 10.8%), however it marginally did not reach statistical significance (p = 0.07). Manual disimpaction was the procedure of choice in the majority of both children (78.3%) and adults (58.6%). Surgical exploration was required more commonly in adults (30% vs 14.5%, p = 0.03). Finally, predisposing factors were more frequently reported in adult than in paediatric cases (24.3% vs 1.2%, p < 0.0001).

Discussion

The word “bezoar” derives its etymology from the Persian word “padzahr” or the Arabic “badizahr”, which both mean “antidote” [1]. Indeed, bezoars had been ascribed mystical powers and were popular through the Middle Ages as remedies against a variety of poisons [1, 6]. It was not until the 19th century that they were recognised as potentially serious medical conditions, being the cause of 3-7% of small intestinal obstructions [7, 8].

Seed bezoars are a distinct subgroup of phytobezoars, caused by the accumulation of indigestible vegetable or fruit seeds in the intestinal lumen. Grains and seeds usually pass through the pylorus and the ileocaecal valve, due to their small size, and accumulate gradually in the colon [3, 4, 9]. Reaching the rectum, the faecal mass is further dehydrated and forms a hard bezoar, commonly presenting as faecal impaction. This pathophysiological process of seed bezoars appears to be different from fibre bezoars, which are usually found in the stomach. Fibres contained in fruits and vegetables (cellulose, lignin, tannins) polymerise and agglutinate in the acidic environment of the stomach, and form a glue-like coagulum which affixes to other material and only rarely overcomes the pyloric sphincter [8, 10].

This hypothesis of different pathophysiology is further supported by the fact that seed bezoars seem to arise in patients without predisposing factors [3, 4]. In our review, previous gastric surgery, neuropsychiatric disease and endocrinopathies (diabetes, hypothyroidism) were recognised only in 12% of cases. On the contrary, retrospective series of gastric and intestinal fibre bezoar cases reported rates of risk factors exceeding 85% [7, 8, 11]. As expected, we observed these predisposing conditions significantly more frequently in adult patients compared to children (24.3% vs 1.2%, p < 0.0001).

Another interesting observation is the geographical distribution of cases, which may reflect the dietary habits across the Mediterranean basin and the Middle East, a diet still including fresh fruits and vegetables, as opposed to the “typical” western diet, based on processed carbohydrates and saturated fats [7]. Watermelons and prickly pears are common delicacies consumed during summer months, while dried sunflower and pumpkin seeds are a favourite snack among all ages and seasons.

As far as the clinical presentation is concerned, the pooled analysis found that seed bezoars occurred most frequently in the rectum, in both children and adults (84% and 70%, respectively). The primary complain was therefore constipation (69% of children, 56% of adults), followed by non-specific abdominal or rectal pain (19% of children, 17% of adults). Intestinal obstruction was relatively rare (17%) and mainly affected those patients with seed bezoars in the terminal ileum. While bowel perforation is the most feared complication, peritonitis was reported only in one case. On the contrary, fibre bezoars in the stomach may run asymptomatic for years or present with vague, non-specific symptoms including epigastric discomfort, abdominal bloating, nausea and vomiting, early satiety, post-prandial fullness, halitosis and weight loss [1].

Diagnosis of seed bezoars should be fairly straightforward, suggested by a careful history. Digital per rectum examination is the sine-qua-non of the diagnostic workup, aided by rectoscopy in selected cases. A full colonoscopy may be advisable in adult patients, to exclude malignant pathology, following bezoar extraction.

On the other hand, seed bezoars in the small intestine and colon are trickier to diagnose and require further investigations. Plain abdominal radiographs may show a solid stool mass, but generally they are within normal limits. Computerised tomography scans are considered the gold standard of diagnosis, offering information about the type, location and degree of obstruction, as well as potential bowel wall ischemia [12, 13]. Of the 20 patients who required supplementary imaging studies, 70% had bezoars proximal to the rectum.

In children with ambiguous right lower quadrant or hypogastric pain, negative imaging studies and suspicion of acute appendicitis, a digital rectal examination should not be omitted. Of 83 children diagnosed with seed bezoars, five had been admitted in hospital with an initial working diagnosis of appendicitis and in three of them a rectal bezoar was revealed, thus eliminating the need for surgery.

Manual evacuation under general anaesthesia is the procedure of choice for rectal seed bezoars, to minimise patient discomfort, while surgery is practically inevitable for small bowel seed bezoars presenting as intestinal obstruction [7]. Indeed, among the 33 cases of operative management in this review, 31 cases concerned seed bezoars of the stomach, duodenum, small and large bowel, proximal to the rectum. Depending on the intraoperative findings, the surgeon may choose between enterotomy and removal of the obstructing bezoar, fragmentation and milking of the bezoar through the ileocaecal valve, and segmental enterectomy.

Only 2/119 (1.7%) rectal seed bezoars required surgical intervention, while only 3/34 (8.8%) gastrointestinal seed bezoars were managed non-operatively with success. Furthermore, whereas manual disimpaction was the most commonly performed therapeutic procedure in both children and adults (78% and 59%, respectively), surgery was more frequently needed in adult patients (30% vs 14.5%, p = 0.03).

Although conservative treatment (endoscopy or chemical dissolution) works well for fibre bezoars, it does not appear to be efficient in patients with seed bezoars [7, 14, 15]. Our review showed that only 6% of seed bezoars were amenable to conservative measures (fleet enemas, stool softeners). Moreover, endoscopy alone usually failed to extract the bezoar, since the endoscope could not pass beyond the seed mass without risking perforation of the rectum, being successful only in 3% of cases.

## Conclusions

In conclusion, seed bezoars should be considered as a distinct subgroup of phytobezoars, with different pathophysiology compared to bezoars caused by fibre accumulation. They are usually found in the rectum of children and adults without predisposing factors, causing constipation and pain. History and digital rectal examination are the cornerstones of diagnosis. In most patients rectal seed bezoars can be manually extracted under general anaesthesia, whereas intestinal seed bezoars are usually found in the terminal ileum causing intestinal obstruction and therefore mandate operative intervention.

## Appendices

**Table 4:** Articles included in the final analysis

1st Author	Year	Country	Journal	Article Data	Title	N
Melchreit	1984	USA	N Engl J Med	310:1748-9	‘Colonic crunch’ sign in sunflower-seed bezoar	1
Cloonan	1988	USA	Ann Emerg Med	17:873-4	Rectal bezoar from sunflower seeds	1
Roberge	1988	USA	Ann Emerg Med	17:131-3	Popcorn Primary Colonic Phytobezoar	1
Dent	1989	USA	Am J Dis Child	143:643-4	Sunflower seed bezoar presenting as diarrhea	1
Meeroff	1989	USA	Am J Gastroenterol	84:650-2	Gastric lupinoma: a new variety of phytobezoar	1
Shah	1990	USA	Pediatr Emerg Care	6:127-8	Polyethylene glycol-electrolyte solution for rectal sunflower seed bezoar	1
Philips	1991	USA	Ann Emerg Med	20:171-2	Sunflower Seed Syndrome: A prickly proctological problem	1
Tsin	1994	Israel	Harefuah	127:227-8	Intestinal obstruction due to lupin phytobezoar in a child	1
Purcell	1995	USA	South Med J	88:87-8	Sunflower seed bezoar leading to fecal impaction	2
Efrati	1997	Israel	J Pediatr Gastroenterol Nutr	25:214-6	Phytobezoar-induced ileal and colonic obstruction in childhood	3
Tsou	1997	USA	Pediatrics	99:896-7	Colonic sunflower seed bezoar	2
Burstein	2000	Israel	Israel Med Assoc J	2:129-31	Small Bowel Obstruction and Covered Perforation in Childhood Caused by Bizarre Bezoars and Foreign Bodies	1
Moons	2000	Netherlands	Ned Tijdschr Geneeskd	144:1878-8	Severe obstipation due to eating unshelled sunflower seeds	2
Lowry	2001	USA	Gastrointest Endosc	53:388-9	Sunflower seed rectal bezoar in an adult	1
Pitiakoudis	2003	Greece	Acta Chir Iugosl	50:131-3	Phytobezoars as a cause of small bowel obstruction associated with a carcinoid tumor of the ileocecal area	1
Sawnani	2003	USA	J La State Med Soc	155:163-4	Proctological crunch: sunflower-seed bezoar	1
Steinberg	2003	Israel	Int J Colorectal Dis	18:365-7	Prickly pear fruit bezoar presenting as rectal perforation in an elderly patient	1
Schoffl	2004	Laos	Asian J Surg	27:348-51	Intestinal obstruction due to phytobezoars of banana seeds: A case report	4
Alexander	2005	India	Indian J Gastroenterol	24:273-4	Cherry pip bezoars causing acute small intestinal obstruction presenting as diabetic ketoacidosis	1
Bakr	2006	UAE	Acta Paediatr	95:886-7	Rectal sunflower seed bezoar	1
Eitan	2006	Israel	Dis Colon Rectum	49:1768-71	Fecal impaction in children: report of 53 cases of rectal seed bezoars	30
Shaw	2007	UK	J Med Case Rep	1:1-4	Large bowel obstruction due to sesame seed bezoar: a case report	1
Singh	2007	Oman	Australas Radiol	51:126-9	Duodenal date seed bezoar: A very unusual cause of partial gastric outlet obstruction	1
Eitan	2007	Israel	J Pediatr Surg	42:1114-7	Fecal impaction in children: report of 53 cases of rectal seed bezoars	53
Bedioui	2008	Tunisia	Gastroenterol Clin Biol	32:596-600	A report of 15 cases of small-bowel obstruction secondary to phytobezoars: Predisposing factors and diagnostic difficulties	8
Kok	2009	Turkey	J Emerg Med	41:537-8	Pumpkin seed bezoar initially suspected as child abuse	1
Mirza	2009	Oman	Trop Doct	39:54-5	Rectal bezoars due to pumpkin seeds	1
Schucany	2009	USA	Proc (Bayl Univ Med Cent)	22:164-5	Abdominal pain, nausea, and vomiting in a 10-year-old girl	1
Kavlakoglu	2010	Turkey	Turkish J Gastroenterol	22:442-3	Very rare coincidence: Perforated duodenal ulcer and olive seed phytobezoar	1
Lane	2010	USA	Pediatr Emerg Care	26:662-4	Sunflower rectal bezoar presenting with an acute abdomen in a 3-year old child	1
Minty	2010	Canada	Can Med Assoc J	182:91991	Rectal bezoars in children	1
Thing	2010	Denmark	Ugeskr Laeger	172:2905-6	Rectal bezoar caused by sunflower seeds	1
Britton	2011	Australia (Iraqi)	J Paediatr Child Health	47:68-9	A case of impacted watermelon seed rectal bezoar in a 12-year-old girl	1
Chauhan	2011	India	Indian J Radiol Imaging	21:21-3	Case report: Colonic bezoar due to Box Myrtle seeds: A very rare occurrence	1
Slesak	2011	Laos	Trop Doct	41:85-90	Bowel obstruction from wild bananas: a neglected health problem in Laos	6
Luporini	2012	Brasil	J Coloproctol	32:308-11	Intestinal obstruction caused by phytobezoar composed of jaboticaba seeds: case report and literature review	1
Manne	2012	USA	Clin Med Res	10:75-7	A Crunching colon: Rectal bezoar caused by pumpkin seed consumption	1
Martinez-Pasqual	2012	Spain	Rev Esp Enferm Dig	104:266-7	Rectal ulcer secondary to a fecal impaction due to pomegranate seed bezoar	1
Al-Rashid	2013	Saudi Arabia	BMJ Case Rep	2013:1-2	Beware of what you eat: small bowel obstruction caused by freekeh bezoars	1
Lim	2013	USA	Endoscopy	45:65212	Unusual cause of constipation: Sunflower seeds bezoar	1
El-Majzoub	2014	Lebanon	Ann Saudi Med	104:2014	Rectal impaction by pomegranate seeds	1
Kia	2014	Iran	West J Emerg Med	15:385-6	Intestinal Obstruction Caused by Phytobezoars	1
Metussin	2014	Brunei	Turkish J Gastroenterol	25:270-1	Rectal bleeding from seeds impaction	1
Plataras	2014	Greece	BMJ Case Rep	2014:1-3	An unusual cause of small bowel obstruction in children: lentil soup bezoar	1
Mahmood	2015	USA	ACG Case Rep J	2:200-1	Rectal Pain and the Colonic Crunch Sign	1
Marchese	2015	Italy	J Biol Regul Homeost Agents	29:707-11	Rectal impaction due to prickly pear seeds bezoar: a case report	1
Akrami	2016	Iran	Iran J Public Health	45:1080-2	Dietary Habits Affect Quality of Life: Bowel Obstruction Caused by Phytobezoar	1
Chai	2016	Malaysia	Indian J Surg	78:326-8	Wild Banana Seed Phytobezoar Rectal Impaction Causing Intestinal Obstruction	1
Nehme	2017	USA	ACG Case Rep J	4:e49	Pumpkin Seed Bezoar Causing Lower Gastrointestinal Bleeding	1
Alexandre	2018	Portugal	BMJ Case Rep	2018	Giant granadilla’s seeds phytobezoar rectal impactation: a very unusual case of intestinal obstruction	1
Islam	2018	Trinidad and Tobago	Int J Surg Case Rep	51:125-9	Mango seed causing acute large bowel obstruction in descending colon-world’s first reported case	1
Manatakis	2018	Greece	Pan Afr Med J	31:157	Rectal seed bezoar due to sunflower seed: a case report and review of the literature	1
